# Observations of blue-stripe artefacts in 2D SWE not reported in guidelines and their influence on diagnostic accuracy—A technical note

**DOI:** 10.1016/j.radcr.2026.06.143

**Published:** 2026-07-17

**Authors:** Marie Byenfeldt, Christer Grönlund, Patrik Nasr, Mattias Ekstedt, Wolf C. Bartholomä, Peter Lundberg, Johan Kihlberg

**Affiliations:** a Clinical Department of Radiology, Region Jämtland Härjedalen, Östersund, Sweden; b Department of Health, Medicine and Caring Sciences, Linköping University, Linköping, Sweden; c Center for Medical Image Science and Visualization (CMIV), Linköping University, Linköping, Sweden; d Department of Diagnostics and Intervention, Umeå university, Umeå, Sweden; e Division of Diagnostics and Specialist Medicine, Linköping University, Linköping, Sweden; f Clinical Department of Radiology, Region Östergötland, Linköping, Sweden; g Clinical Department of Medical Radiation Physics, Region Östergötland, Linköping, Sweden

**Keywords:** Liver diseases, Ultrasonography, Elasticity imaging techniques, Magnetic resonance elastography, Acoustic radiation force impulse imaging, Artifacts

## Abstract

Two-dimensional shear wave elastography is a clinically established technique used to detect and stage liver fibrosis. It generates a real-time elastogram map, which visually displays tissue stiffness and assists in assessing the severity of liver fibrosis. The elastogram map serves as a graphical depiction of how stiff or soft the liver tissue is, providing valuable information for diagnosis. However, artefacts can appear in elastogram maps. In this work, we report on “blue-stripe” artefacts that can appear in elastogram maps without being indicated as a technical error by the system and that are not mentioned in guidelines. To the best of our knowledge, this type of artefact has not been described in the literature and may inadvertently be included in measurements, potentially leading to inaccurate results. We describe 4 cases in which a blue-stripe artefact was present on liver 2-dimensional shear wave elastography elastograms and compare these measurements with artefact-free 2-dimensional shear wave elastography acquisitions and magnetic resonance elastography. In all 4 cases, 2-dimensional shear wave elastography values obtained in the presence of the blue-stripe artefact were lower than the corresponding artefact-free 2-dimensional shear wave elastography values and lower than expected from magnetic-resonance-elastography–based fibrosis assessment, creating a risk of underestimating advanced fibrosis. These cases highlight the importance of real-time recognition of blue-stripe artefacts during liver stiffness assessment. Excluding artefact-affected acquisitions may improve measurement reliability and help avoid false low-stiffness values in routine clinical practice.

## Introduction

Liver fibrosis has various etiologies and may progress from fibrosis to cirrhosis, ultimately leading to hepatocellular carcinoma (HCC) [1]. The global burden of chronic liver diseases is large and growing [[2], [3], [4]]; therefore, reliable noninvasive methods to detect, stage, and follow up on liver fibrosis are of the utmost importance.

The first ultrasound method available to assess liver stiffness was transient elastography (TE) (Echosens, Paris, France), which generates shear waves at the skin surface through mechanical excitation. Because TE does not provide concurrent imaging, the operator cannot select the depth or exact area of measurement within the liver parenchyma [5]. This 1-dimensional technique was later developed into the 2-dimensional (2D) shear wave elastography (SWE) imaging method, in which acoustic radiation force impulses (ARFIs)—that is, “push” pulses—create shear waves at a certain depth in the liver parenchyma, as selected by the ultrasound operator. The 2D SWE method was commercially introduced in 2009 with the Aixplorer ultrasound system (SuperSonic Imagine S.A., Aix-en-Provence, France) [6]; it has shown higher diagnostic accuracy than TE and correlates well with liver biopsy for fibrosis staging [7,8]. Liver stiffness can also be assessed using magnetic resonance elastography (MRE), which has demonstrated high diagnostic accuracy [9,10], although its availability remains limited.

Several factors are associated with less-reliable liver stiffness measurements on 2D SWE, including older age, higher body mass index, male sex [11], and increased skin-to liver capsule distance [12,13]. It is also important to understand how different artefacts may affect 2D SWE measurements in clinical practice [14]. Early guidelines mention several types of artefacts, although these are described mainly for strain elastography [15]. Updated guidelines briefly address artefacts in liver elastography (primarily reverberation artefacts) and do not provide illustrative examples [16]. Some ultrasound devices with 2D SWE include an elastogram map, provided as a real-time, color-coded visual representation of the use of tissue stiffness to assess liver fibrosis [17]. However, the appearances of different artefacts on an elastogram are not widely recognized among 2D SWE operators. One such artefact in routine clinical practice is the blue-stripe artefact, which typically results from patient or probe movement. To the best of our knowledge, this type of artefact is not described in guidelines, and its imaging characteristics and quantitative effect on 2D SWE measurements remain poorly described, potentially leading to inaccurate results.

The aim of this case series was to compare liver 2D SWE measurements obtained in the presence of blue-stripe artefacts with artefact-free 2D SWE measurements, using MRE as a reference standard.

## Case presentation

### Ultrasound measurements

The 2D SWE measurements were obtained using a GE LOGIQ E10 rev 4 ultrasound system (General Electric Healthcare, Seoul, Korea) with a convex probe (C1-[6]) and comb-push technology, a technology that makes it possible to increase the energy output by activating the penetration mode and thereby increasing the terminal index [18]. In comb-push technology, several simultaneous push pulses arranged spatially in the image are used to compensate for the reduction in amplitude as the waves propagate [[19], [20], [21]]. A time-of-flight algorithm was used to estimate the local shear wave speed at every location in the SWE region of interest (ROI). Thus, the shear wave speed was expressed in meters per second, but the speed was converted into the Young’s modulus. Measurements were acquired during end-expiration breath holds via an intercostal approach.

The ultrasound display was divided into 2 images, with a quality map to the left and the elastogram map to the right. The upper border of the elastogram map was placed about 2 cm beneath the Glisson capsule to avoid reverberation artefacts [17], and the size of the ROI was fixed at 1.0 cm. A red ROI indicates no successful technical measurements with deficient shear waves. The angel of the beam was maintained at zero and the probe was held perpendicular to the liver capsule [22]. 2D SWE measurements were performed according to the European Federation of Societies for Ultrasound in Medicine and Biology (EFSUMB) guidelines in supine body position [17].

A 2D SWE measurements ≥9 kPa correspond to liver fibrosis stage F3, with a suggestion of compensated advanced chronic liver disease [16,23,24].

### Magnetic resonance imaging measurements

Magnetic resonance imaging proton density fat fraction (PDFF) and MRE were performed on a 3-T Ingenia system (Philips Healthcare, Best, The Netherlands). The PDFF covered the entire liver [25] and was used for the evaluation of hepatic steatosis, defined as ≥5% PDFF. An acoustic transducer (Resoundant, Rochester, MN) operating at 60 Hz was used, with the amplitude adjusted for body weight, and acquisitions were obtained during single 13-second breath-holds. Elastogram maps were generated using the vendor’s software. For MRE, a liver stiffness value of 3.7 kPa corresponded to fibrosis stage ≥F3 [9,10].

To ensure an exact comparison between the MRE and 2D-SWE images, measurements for both methods were collected in liver segment VIII. The ROI size for the 2D SWE was set at 1.0 cm; to match this, the MRE ROI was estimated at 79 mm².

### Representative 2D SWE artefacts observed in patients without advanced fibrosis

**Fig. 1:**
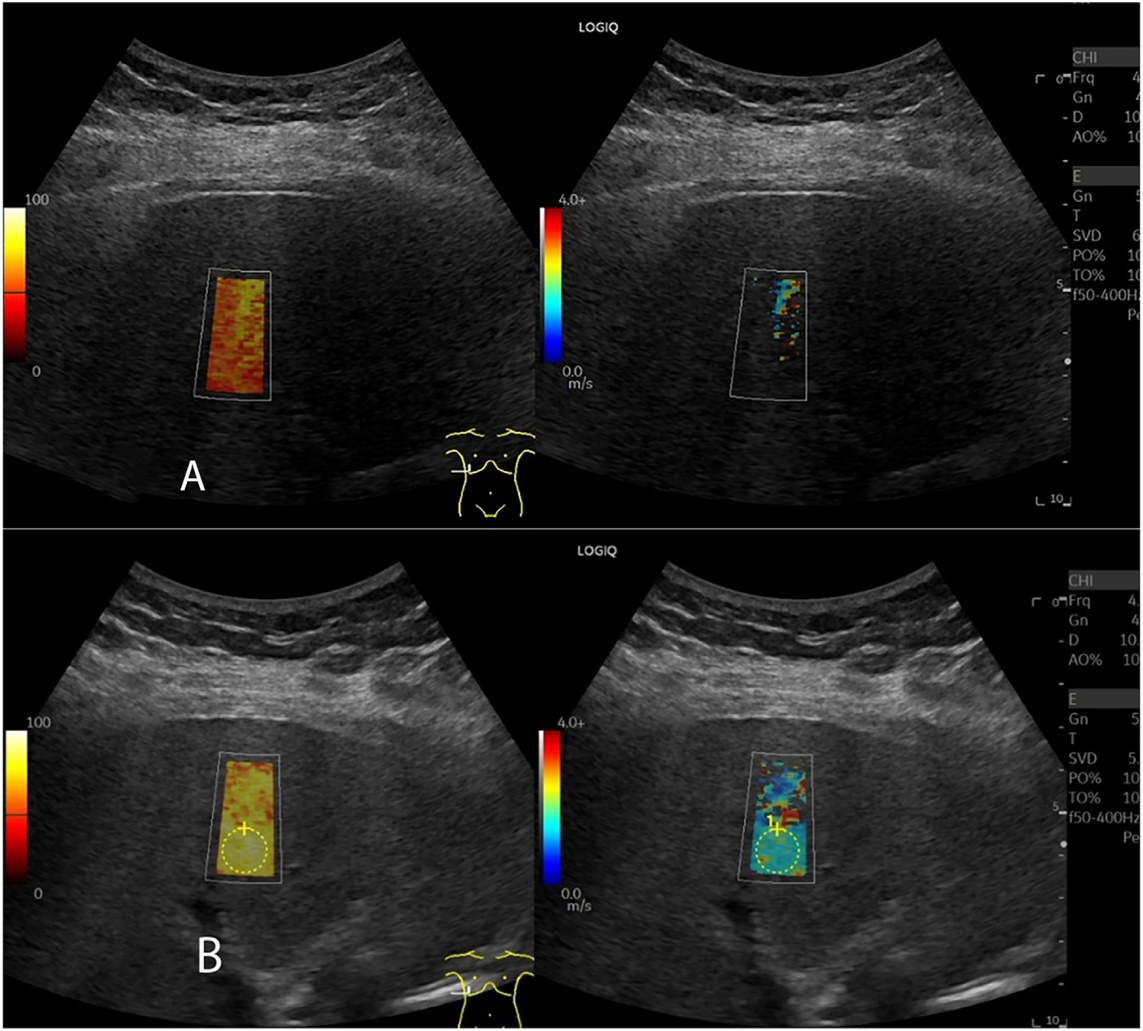
(A, B) Representative 2D SWE artefacts in a patient without advanced fibrosis. The quality map is shown on the left and the elastogram on the right. (A) Rib shadowing; (B) a reverberation artefact affecting the superficial elastogram; the deeper portion remains suitable for measurement, as indicated by the quality map and yellow ROI.

Fig. 1A and B shows representative 2D SWE artefacts consisting of rib shadowing (Fig. 1A) and a reverberation artefact (Fig. 1B) in a patient without advanced fibrosis. In both examples, the quality map on the left demonstrates a reduced signal. As shown in Fig. 1B, the deeper portion of the elastogram below the reverberation artefact remains suitable for measurement, as supported by the quality map and the yellow ROI.

**Fig. 2:**
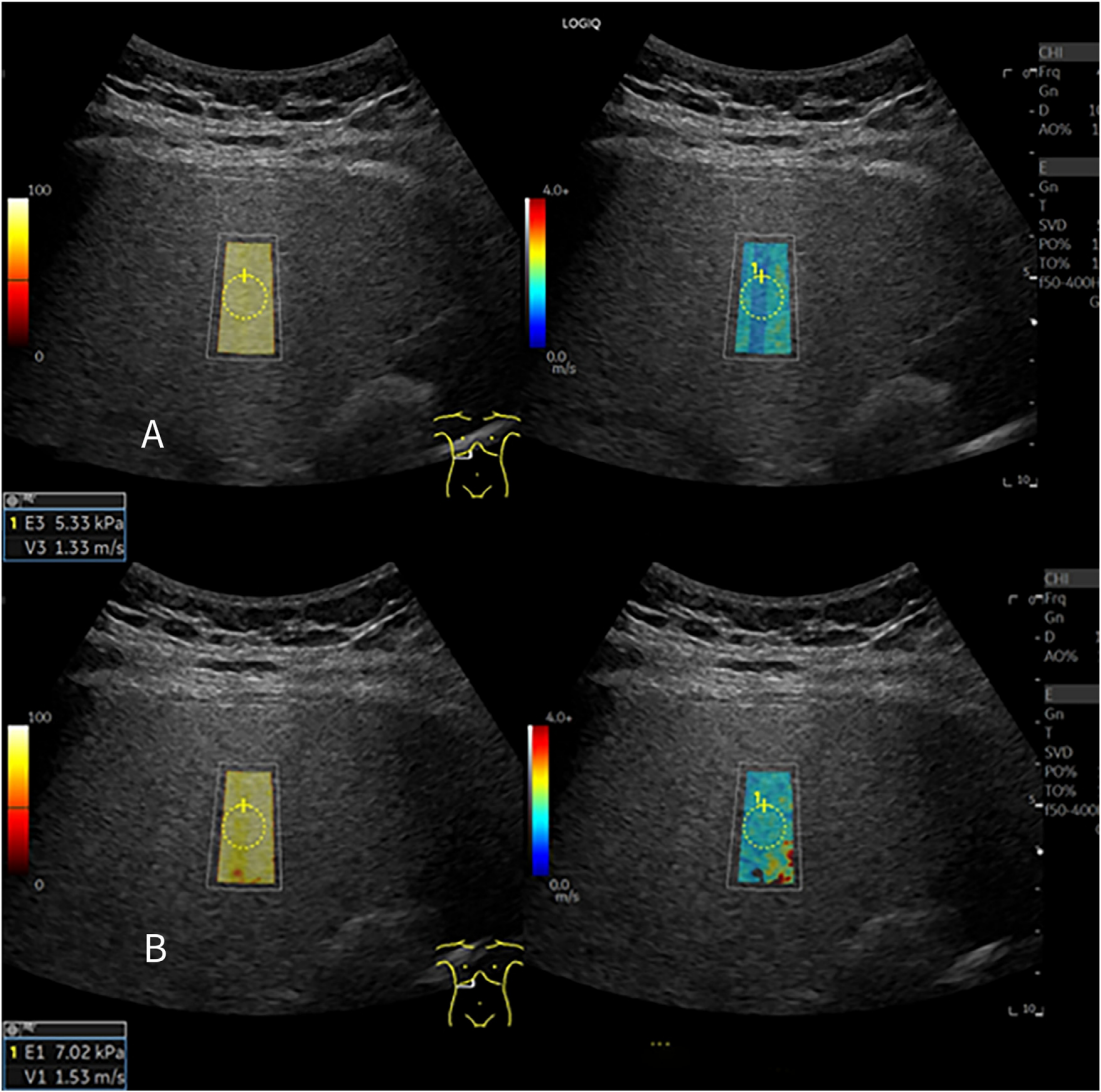
(A, B) 2D SWE images from a patient without advanced fibrosis. The quality map is shown on the left and the elastogram on the right. (A) A blue-stripe artefact is present, despite the preserved measurement quality indicated by the quality map and yellow ROI. (B) an artefact-free acquisition from the same patient.

Fig. 2A and B shows 2D SWE images from another patient without advanced fibrosis. A blue-stripe artefact is present in Fig. 2A and absent in Fig. 2B. The quality map indicates preserved measurement quality, with a yellow ROI in both images. Despite this, the 2D SWE value is lower in the presence of the blue-stripe artefact.

Table 1 compares 2D SWE images with and without blue-stripe artefacts, along with MRE images as references, in 4 different patients with advanced fibrosis.

**Table 1:** Comparison of liver stiffness measurements obtained with 2D SWE in the presence and absence of a blue-stripe artefact, with MRE as a reference.

	2D SWE measurements with blue-stripe artefact	2D SWE measurements without blue-stripe artefact	MRE measurements	Fibrosis grade ≥F3
Case 1	6.7 kPa	10.2 kPa	8.4 kPa	yes
Case 2	5.0 kPa	9.3 kPa	5.6 kPa	yes
Case 3	6.7 kPa	12.0 kPa	6.2 kPa	yes
Case 4	6.0 kPa	18.3 kPa	9.7 kPa	yes

2D SWE, two-dimensional shear wave elastography; MRE, magnetic resonance elastography

**Fig. 3:**
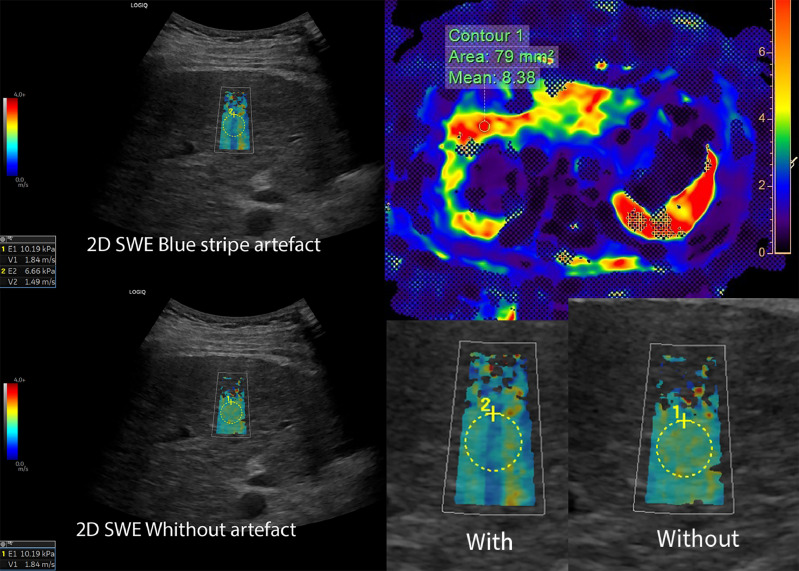
(Left) 2D SWE and (right) MRE image of the first case. In the top left image, a vertical dark blue stripe crosses the elastogram map, and the SWE result is 6.7 kPa. In the bottom left image, which has no artefact, the SWE result is 10.2 kPa, which corresponds with the MRE of 8.4 kPa (top right side) and more consistent with advanced fibrosis.

In the first case, shown in Fig. 3, the penetration mode was activated. The distance between the liver capsule and the probe was 2.5 cm, and no steatosis was present. The top left image shows a vertical blue stripe artefact across the elastogram map, with a 2D SWE measurement of 6.7 kPa. The bottom left image represents the same case without the artefact and demonstrates an increased 2D SWE measurement of 10.2 kPa. Compared with the MRE value of 8.4 kPa, the higher 2D SWE value appears more consistent with advanced fibrosis. These findings suggest that the blue-stripe artefact has produced an artificially low 2D SWE measurement.

**Fig. 4:**
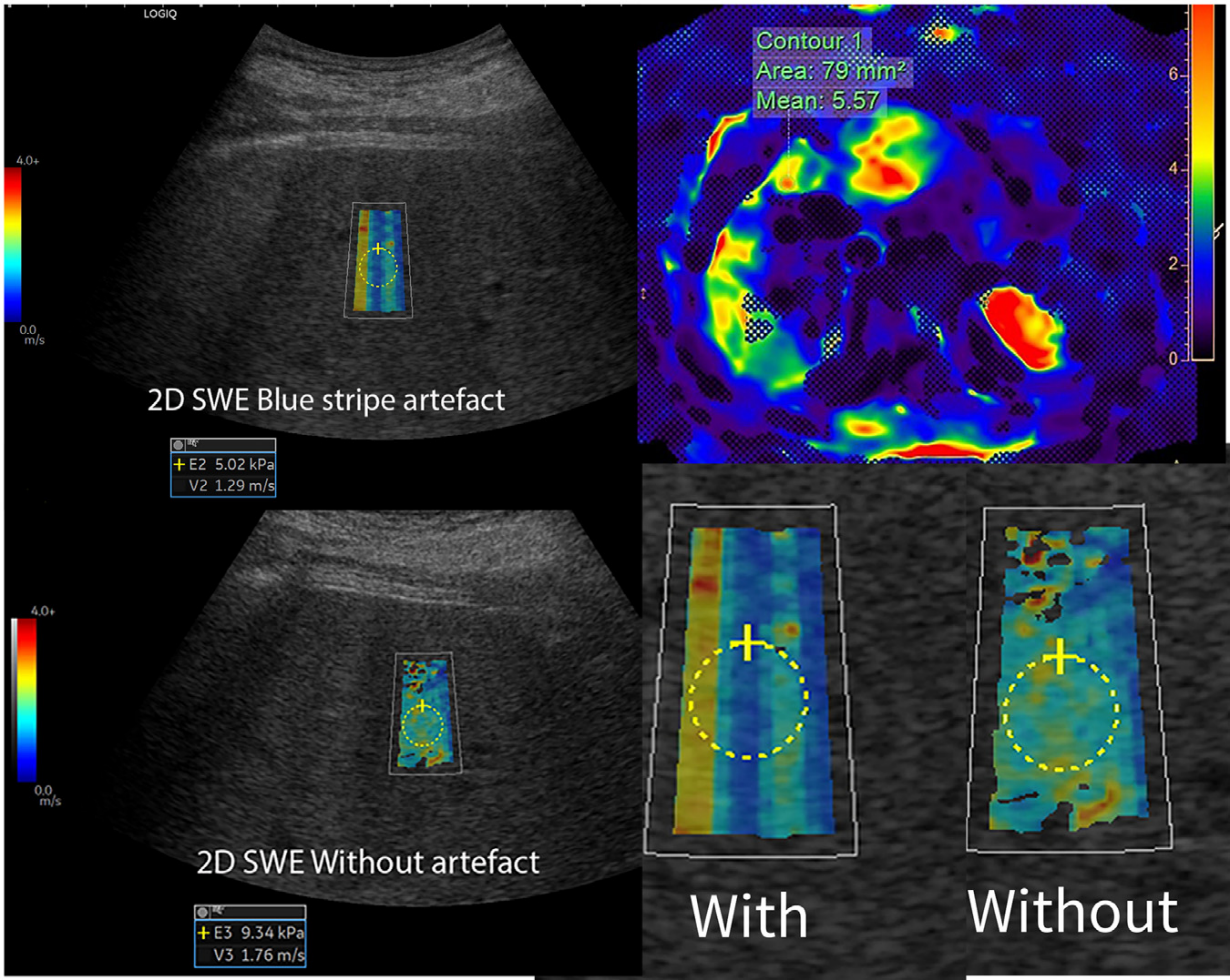
(Left) 2D SWE and (right) MRE image of the second case. In the top left image, a vertical dark blue stripe crosses the elastogram map, and the SWE result is 5.0 kPa. In the bottom left image, which has no artefact, the SWE result is 9.3 kPa, which corresponds with the MRE result of 5.6 kPa (top right side) and more consistent with advanced fibrosis.

In the second case, shown in Fig. 4, the penetration mode was activated, and the distance between the liver capsule and the probe was 2.3 cm. No hepatic steatosis was present. The top left image, which has a very clear vertical blue-stripe artefact across the elastogram map, gives a 2D SWE measurement of 5.0 kPa. In the image with no artefact (bottom left), a higher 2D SWE value of 9.3 kPa is obtained. Compared with the MRE value of 5.6 kPa, the higher 2D SWE value again appears more consistent with advanced fibrosis. Accordingly, the blue-stripe artefact appears to have produced a falsely low 2D SWE value.

**Fig. 5:**
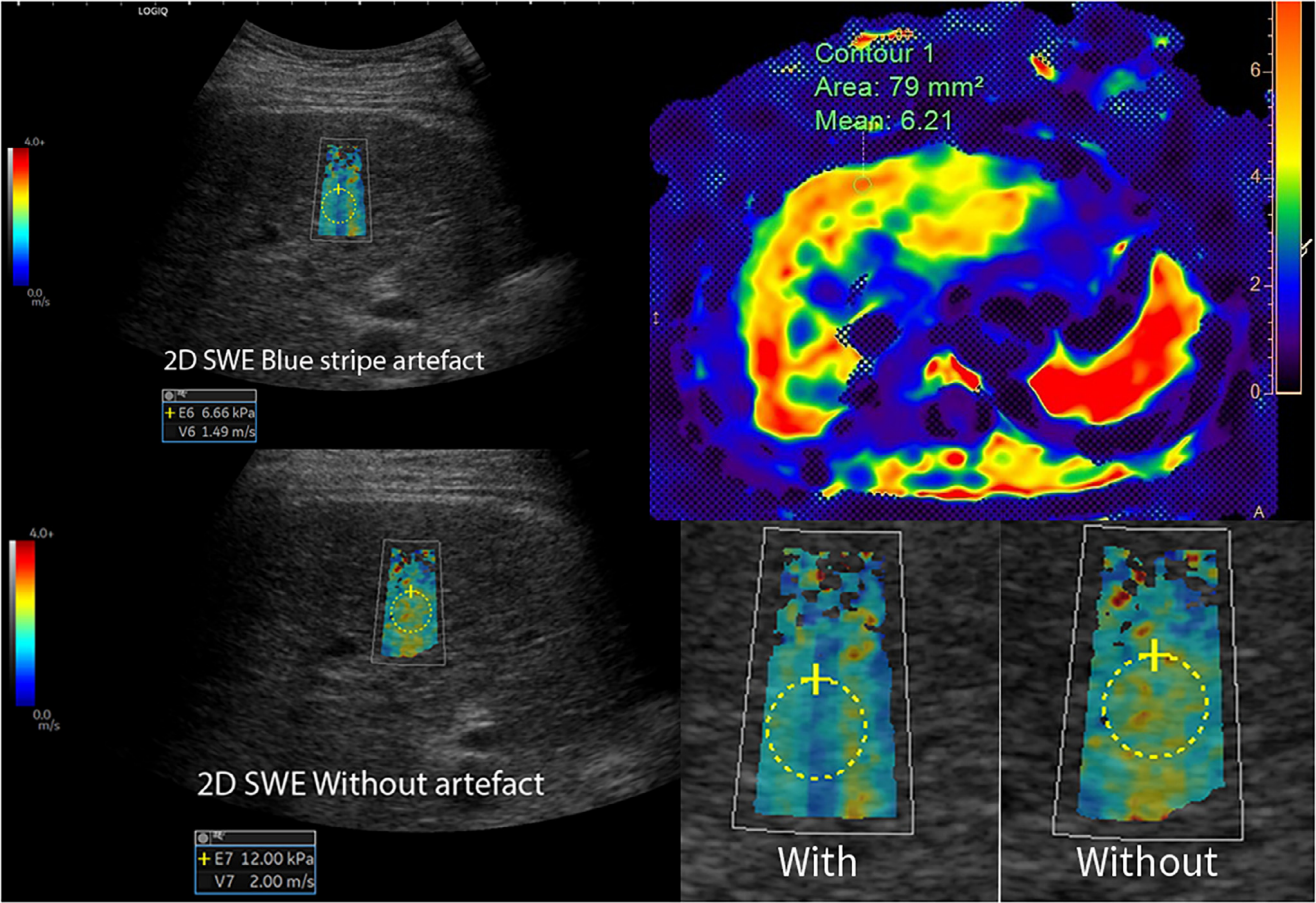
(Left) 2D SWE and (right) MRE image of the third case. In the top left image, a thin blue vertical dark blue stripe crosses the elastogram map, resulting in an SWE result of 6.7 kPa. In the bottom left elastogram map, which has no artefact, the SWE result is 12.0 kPa, which corresponds with the MRE of 6.2 kPa (top right side) and more consistent with advanced fibrosis.

In the third case, shown in Fig. 5, the penetration mode was not activated, and the distance between the liver capsule and the probe was 1.8 cm. The patient had no hepatic steatosis. A narrow blue vertical artefact is visible across the elastogram map depicted in the top left image, and the 2D SWE measurement for this image is 6.7 kPa. In the elastogram map with no artefact (bottom left in Fig. 5), the 2D SWE value increases to 12.0 kPa. Compared with the MRE value of 6.2 kPa, the higher 2D SWE value appears more consistent with advanced fibrosis. Thus, the blue-stripe artefact has produced a false-low 2D SWE measurement.

**Fig. 6:**
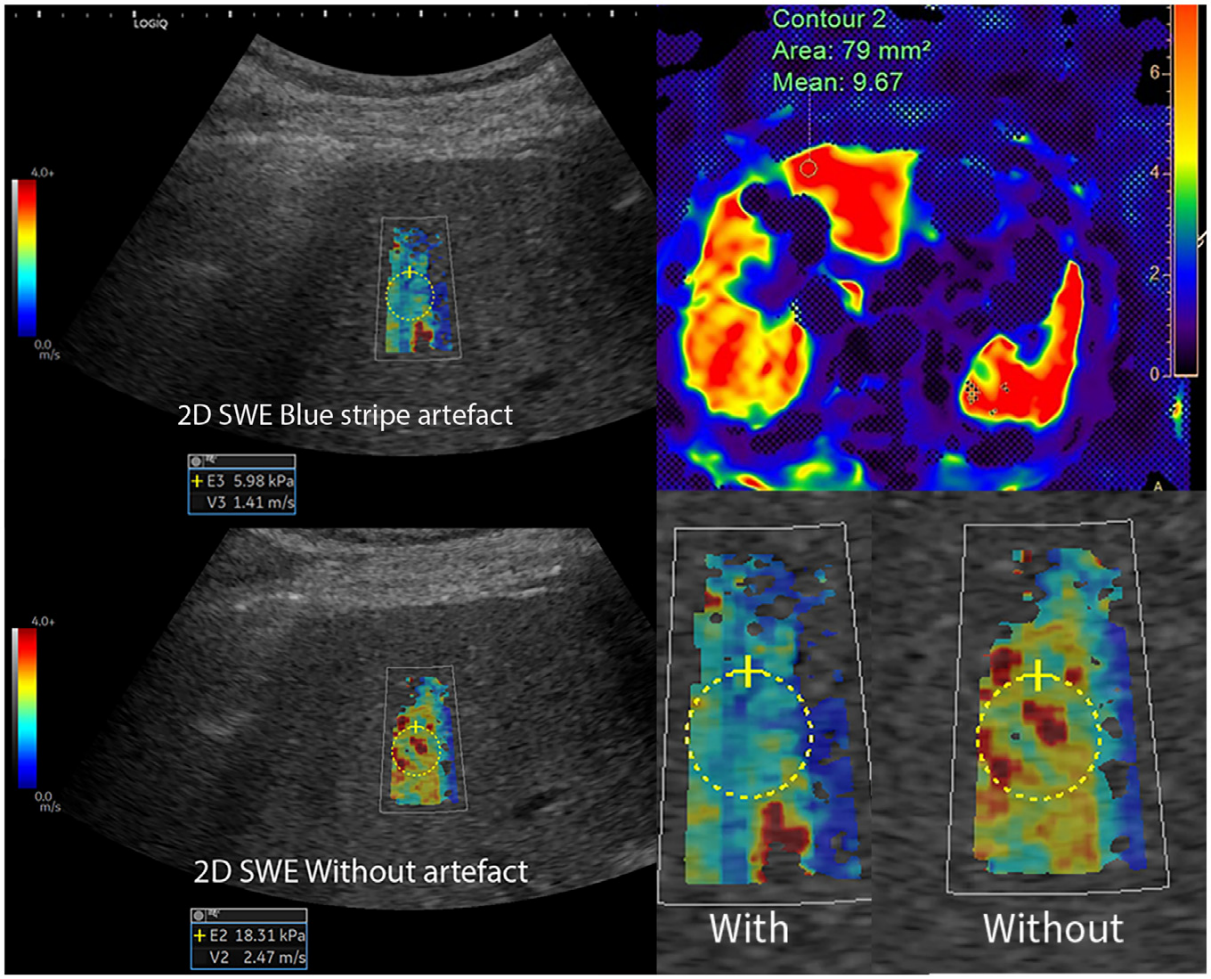
**.** (Left) 2D SWE and (right) MRE image of the fourth case. In the top left image, a vertical dark blue stripe crosses the elastogram map; the image yields an SWE result of 6.0 kPa. In the bottom left image, which has no artefact in the elastogram map, the SWE result is as high as 18.3 kPa and corresponds with the MRE of 9.7 kPa (top right side) and more consistent with advanced fibrosis.

In the final case, Fig. 6, the penetration mode was activated. The distance between the liver capsule and the probe was 1.7 cm, and hepatic steatosis was present. The upper left image shows a vertical blue-stripe artefact on the elastogram, with a 2D SWE measurement of 6.0 kPa. The lower left image, obtained without an artefact, shows a higher 2D SWE measurement of 18.3 kPa. Compared with the MRE value of 9.7 kPa, the higher 2D SWE value appears more consistent with advanced fibrosis. These findings indicate that the blue-stripe artefact has led to a falsely low 2D SWE measurement.

## Discussion

To ensure adequate measurement quality in clinical practice, clinicians depend on quality maps and ROI appearance. The quality map provides guidance regarding image suitability for measurements; in addition, a yellow ROI indicates that the measurement quality is adequate, while a red ROI indicates unsuccessful technical measurements due to insufficient shear wave generation, such that measurements are not advised.

This technical report presents 4 cases from a clinical study, demonstrating a blue vertical artefact stripe across the elastogram map. In these 4 cases involving patients with advanced fibrosis, images exhibiting blue-stripe artefacts in the elastogram maps displayed a yellow ROI, and the quality map verified measurement adequacy.

These findings suggest that the blue-stripe artefact can artificially lower 2D SWE measurements. This interpretation is supported by a comparison with MRE-derived liver stiffness values in our case report. The observed pattern indicates that the artefact is associated with falsely low stiffness estimates rather than true biologic variation. Failure to recognize this artefact may lead clinicians to accept affected measurements, with a consequent risk of underdiagnosing advanced fibrosis.

To the best of our knowledge, this artefact has not been described in the literature. Our observations are in line with prior work showing that motion can reduce liver stiffness measurements on SWE [26], although that study did not include a reference standard.

Reliable elastography depends on adequate underlying B-mode image quality and stable motion tracking. As in conventional sonography, image quality is influenced by the spatial, contrast, and temporal resolution, and suboptimal conditions may degrade measurement performance. In practice, optimization of the acoustic window, signal-to-noise ratio, output settings, and operating frequency may improve elastogram quality and reduce susceptibility to error. Recognition of visually abnormal elastogram patterns is therefore an important component of measurement quality control. Ultrasound artefacts and noise remain important sources of diagnostic error [27,28]. For liver 2D SWE, familiarity with ultrasound physics, recognition of common artefacts, and adherence to acquisition technique are essential for reliable measurements. These competencies contribute to improved patient safety and diagnostic accuracy. Additionally, operators should ensure that the ultrasound equipment is sufficiently modern and not outdated [29,30]. Current recommendations emphasize operator experience as well, with suggested thresholds of more than 300 abdominal ultrasound examinations or more than 50 supervised 2D SWE examinations [17], which may be particularly relevant when subtle elastogram artefacts are present.

2D SWE is based on ARFI technology, in which focused ultrasound pushes generate shear waves that are tracked using time-of-flight methods [31,32]. Because stiffness estimation depends on accurate motion tracking, the method is inherently sensitive to factors that disturb wave propagation or tracking fidelity [33]. Known limitations of liver 2D SWE include reverberation beneath the liver capsule, respiratory motion, cardiac motion, vascular pulsation, and focal signal loss [14]. However, the visual appearance and practical interpretation of these artefacts are not well illustrated in current guidance [16,17]. This may be especially relevant across different vendor platforms, which vary in acquisition schemes and quality-assurance displays. Additionally, various technologies exist for 2D SWE liver evaluations, each offering differing quality and reliability measurement programs.

A plausible explanation for the blue-stripe artefact is motion-related disruption of shear wave generation or tracking caused by respiration, body movement, probe instability, or vascular pulsation. In GE comb-push 2D SWE, repeated push beams and interleaved tracking may be particularly vulnerable to such disturbances, producing localized modulus abnormalities on the elastogram. From a practical standpoint, the elastogram itself may serve as a real-time reliability cue, and recognition of a blue-stripe pattern should prompt reacquisition rather than acceptance of the recorded value [34].

## Conclusion

Blue-stripe artefacts on GE comb-push 2D SWE elastogram maps may result in falsely low liver stiffness measurements and thus lead to underestimation of advanced fibrosis. In this case series, artefact-affected acquisitions yielded lower stiffness values than artefact-free 2D SWE measurements and were less concordant with MRE results. Careful real-time inspection of the elastogram map, with rejection of acquisitions containing a blue-stripe artefact, may improve measurement reliability in clinical practice. Further studies across larger cohorts and vendor platforms are needed to clarify the mechanism, prevalence, and clinical impact of this artefact.

## Data availability statement

All data are shared in this technical note. Data and materials supporting the results or analyses presented in this paper will be made available upon reasonable request.

## Ethical approval

This study was approved by the Swedish Ethical Review Authority (No. 2022-01610-01) and performed in accordance with the 2013 Declaration of Helsinki. Data was collected from September to December 2022. Written informed consent for the publication of this case report and the accompanying images was obtained from all participants. The radiological images contain no identifiable information.

## Patient consent

All study participants provided written informed consent for publication of their cases. The radiological images contain no identifiable information.
